# Selection of reliable reference genes for quantitative real-time PCR gene expression analysis in Jute (*Corchorus capsularis*) under stress treatments

**DOI:** 10.3389/fpls.2015.00848

**Published:** 2015-10-14

**Authors:** Xiaoping Niu, Jianmin Qi, Gaoyang Zhang, Jiantang Xu, Aifen Tao, Pingping Fang, Jianguang Su

**Affiliations:** ^1^Key Laboratory for Genetics, Breeding and Multiple Utilization of Crops, Fujian Agriculture and Forestry UniversityFuzhou, China; ^2^College of Life Sciences, Shangrao Normal UniversityShangrao, China; ^3^Institute of Bast Fiber Crops, Chinese Academy of Agricultural SciencesChangsha, China

**Keywords:** jute (*Corchorus capsularis*), qRT-PCR, reference genes, gene expression, abiotic and biotic stress

## Abstract

To accurately measure gene expression using quantitative reverse transcription PCR (qRT-PCR), reliable reference gene(s) are required for data normalization. *Corchorus capsularis*, an annual herbaceous fiber crop with predominant biodegradability and renewability, has not been investigated for the stability of reference genes with qRT-PCR. In this study, 11 candidate reference genes were selected and their expression levels were assessed using qRT-PCR. To account for the influence of experimental approach and tissue type, 22 different jute samples were selected from abiotic and biotic stress conditions as well as three different tissue types. The stability of the candidate reference genes was evaluated using geNorm, NormFinder, and BestKeeper programs, and the comprehensive rankings of gene stability were generated by aggregate analysis. For the biotic stress and NaCl stress subsets, *ACT7* and *RAN* were suitable as stable reference genes for gene expression normalization. For the PEG stress subset, *UBC*, and *DnaJ* were sufficient for accurate normalization. For the tissues subset, four reference genes *TUB*β, *UBI, EF1*α, and *RAN* were sufficient for accurate normalization. The selected genes were further validated by comparing expression profiles of *WRKY15* in various samples, and two stable reference genes were recommended for accurate normalization of qRT-PCR data. Our results provide researchers with appropriate reference genes for qRT-PCR in *C*. *capsularis*, and will facilitate gene expression study under these conditions.

## Introduction

With the increase of global environmental awareness, more and more people are actively purchasing goods made from ecologically friendly materials. Unfortunately, many of these materials are intrinsically unrecyclable, including many of the predominantly used polymers. However, many natural fibers such as jute and kenaf possess properties that are comparable to more traditional composites, including stiffness, flexibility, impact resistance, and elasticity (Sydenstricker et al., 2003). The overlap between natural fiber properties and those of traditional, reinforced composites are underscored by their environmentally friendly nature. For instance, fiber components are biodegradable, renewable, and result in low energy consumption. Collectively, these characteristics lend natural fibers to being logical substitutes for non-renewable synthetic fibers (Oksman et al., 2003; Corrales et al., 2007).

Jute (*Corchorus capsularis* L.) is an annual herbaceous fiber crop. It is found predominantly in Southeast Asia and is the second cheapest and most commercially available fiber crop, whereby it provides a biodegradable and renewable lignocellulose fiber. Jute is a promising and featured fiber material among many value-added industrial products, due in large part to its high luster, moisture absorption properties, ability for rapid water loss, and easy breakdown. Furthermore, jute has much potential for use in the industrial production of packaging materials (Sydenstricker et al., 2003). With the recent and increasing interest in jute, work has been conducted to better understand its physiological and biochemical properties (Corrales et al., 2007; del Río et al., 2009; Defoirdt et al., 2010). At a molecular level, several studies have also been done to examine relevant molecular markers (e.g., SSR, ISSR, RAPD, and AFLP) (Qi et al., 2003a,b; Basu et al., 2004; Roy et al., 2006; Mir et al., 2008, 2009), ESTs, stress response factors (Alam et al., 2010), and a transformation system (Chattopadhyay et al., 2011; Zhang et al., 2013). However, many of these previous experiments used single or multiple gene expression analysis via classical RT-PCR and/or Northern blot (Alam et al., 2010). Only a handful of studies have used quantitative reverse transcription PCR (qRT-PCR) to determine the expression pattern of functional jute genes (Zhang et al., 2013, 2014).

As of this writing, qRT-PCR provides the most efficient, sensitive, low cost, and reproducible method for accurate and rapid detection and quantification of mRNA transcription levels for a given gene of interest (Bustin, 2002). However, several factors including RNA integrity, reverse transcription efficiency, cDNA quality, sample amount, and/or extraneous tissue and cell activities can significantly influence the accuracy of gene expression (Bustin, 2002; Huggett et al., 2005). To lessen these problems, one or more reference genes are required to account for the variance between samples and/or reactions. To this end, the transcription level of an ideal reference gene should remain constant across different tissues, treatments, and developmental stages (Gutierrez et al., 2008). A number of commonly used housekeeping genes (e.g., β-actin, elongation factor 1α, 18S ribosomal RNA, and polyubiquitin) have been used, but results indicated that their expression levels are somewhat unstable. Thus, their use as internal reference genes should be taken with some caution under a given set of experimental conditions (Gutierrez et al., 2008). Taken together, these inconsistencies highlight the need to evaluate the stability of candidate reference genes under the relevant experimental conditions prior to using for gene expression normalization by qRT-PCR.

In recent years, an increasing number of stable reference genes have been studied in several systems, including eukaryotic elongation factor 1-alpha (*EF1*α) and F-box family protein (*FBX*) in *Arabidopsis thaliana*; 18S ribosomal RNA (18S *rRNA*), ubiquitin5 (*UBQ5*), and eukaryotic elongation factor 1-alpha (*eEF1*α) in *Oryza sativa* (Czechowski et al., 2005; Jain et al., 2006; Remans et al., 2008; Narsai et al., 2010); catalytic subunit of protein phosphatase 2A (*PP2A*), actin 4 (*ACT4*), ubiquitin 14 (*UBQ14*), F-box family protein (*FBX6*), clathrin adaptor complexes medium subunit family protein (*MZA*), and polypyrimidine tract-binding protein homolog (*PTB*) in *Gossypium hirsutum* (Artico et al., 2010). Furthermore, Zhang et al. and Saraiva et al. both found that *EF1*α could also be used as a reference gene in *Triticum aestivum* (Paolacci et al., 2009; Long et al., 2010) as well as in *Glycine max* (Jian et al., 2008; Libault et al., 2008). Additionally, suitable reference genes for use in gene expression analysis have also been methodically identified for a variety of species, such as *Saccharum officinarum* (Ling et al., 2014), *Litchi chinensis* (Zhong et al., 2011), *Litsea cubeba* (Lin et al., 2013), *Citrus sinensis* (Mafra et al., 2012), *Coffea* ssp. (Cruz et al., 2009), *Solanum tuberosum* (Nicot et al., 2005), *Musa acuminata* (Chen et al., 2011), *Vitis vinifera* (Reid et al., 2006), *Helianthus annuus* (Fernandez et al., 2011), *Nicotiana tabacum* (Schmidt and Delaney, 2010; Liu et al., 2012), and *Linum usitatissimum* (Huis et al., 2010). However, no systematic validation of reference genes has been performed in jute (*Corchorus capsularis*), which prevents further studies on this species at the functional gene expression and transcriptome profile analysis levels.

In the present study, 11 genes were selected as candidate reference genes for evaluation expression stability in jute: 18S ribosomal RNA (18S *rRNA*), actin (*ACT*), actin 7 (*ACT7*), chaperone protein dnaJ (*DnaJ*), eukaryotic elongation factor 1-alpha (*EF1*α), ras-related small GTP-binding protein (*RAN*), alpha-tubulin (*TUB*α), beta-tubulin (*TUB*β), ubiquitin-conjugating enzyme like protein (*UBC*), ubiquitin extension protein (Imai et al., 2014) and ubiquitin (*UBQ*). We then sought to reveal which reference gene(s) were the best option for gene expression qRT-PCR analysis of jute under various experimental treatments using three available statistical algorithms, geNorm (Vandesompele et al., 2002), NormFinder (Andersen et al., 2004), and BestKeeper (Pfaffl et al., 2004). Finally, we validated the expression levels of the transcription factor *CcWRKY15* using the selected reference gene(s). The results from this work will facilitate future studies on gene expression as well as foster a better understanding of how novel genes function in the molecular mechanisms of jute biological and/or physiological processes.

## Materials and methods

### Plant materials and treatments

Jute (*Corchorus capsularis* L.) variety Huangma 108 was used for all experimental treatment groups. To ensure disease-free materials, seeds were rinsed under running water for 10 min and sterilized with 5% sodium hypochlorite for 10 min. They were then washed three times with sterile water before germinated on filter paper that had been saturated with water in complete darkness at 28°C. After 3 days, seedlings were grown in the greenhouse in 1/4 Hoagland solution under a 16/8-h light/dark cycle at 30/26°C (day/night). Seedlings were assessed at the 3–5 leaf stage and the most consistent were used for (i) the abiotic and biotic stress groups or for (ii) harvesting of different plant tissues (e.g., root, stem, and leaf).

For the salinity and drought treatments, seedlings were subjected to 200 mM sodium chloride and 15% (w/v) PEG6000, respectively, and harvested at 0, 2, 4, 6, 8, 12, and 24 h. For the biotic treatment, seedlings were inoculated with 2 ml (10^6^ spores per ml) of *Colletotrichum siamense* spores in suspension previously described by Ma et al. (2009) and then sampled at 0, 1, 6, 12, 24, 48, 72, and 96 h. Tissues from the roots, stems, and leaves were collected from plants in the 3–5 leaf stage that had grown well under the experimental conditions. All samples were harvested from three replicate plants, giving a total of 25 samples comprised of 14 abiotic and eight biotic stress treatment samples and three tissue-specific samples. Samples were immediately frozen in liquid nitrogen and stored at −80°C until RNA extraction.

### Total RNA extraction and cDNA synthesis

Total RNA was extracted from approximately 100 mg fresh leaf tissue using the OMEGA isolation kit (R6827-01, USA) according to the manufacturer's instructions. The genomic DNA contamination was eliminated using RNase-free DNase I (TaKaRa, Japan) and RNA sample quality was then determined using the NanoDrop 2000 spectrophotometer (NanoDrop, Thermo Scientific). RNA integrity was assessed by 2.0% agarose gel electrophoresis. Finally, RNA samples with an A_260_/A_280_ ratio between 1.9 and 2.1 and an A_260_/A_230_ ratio greater than 2.0 were used for further analysis. Subsequently, the first-strand cDNA was synthesized from 1 μg total RNA in a volume of 20 μl using the PrimeScript® RT reagent kit (TaKaRa, Japan) by following the manufacturer's protocol. All final cDNA samples were diluted 10-fold for subsequent quantitative real-time RT-PCR reactions. Samples were stored at −20°C until use.

### Primer design, verification of PCR products, and qRT-PCR

A total of 11 candidate reference genes, including 18S *rRNA* (FJ527599), *ACT* (GU207477), *ACT7* (GH985158), *DnaJ* (GR463675), *EF1*α (GH985217), *RAN* (FK826494), *TUB*α (GH985177), *TUB*β (JK743820), *UBC* (GR463733), *UBI* (GH985256), and *UBQ* (FK826547), were identified after performing a TBLASTN among the jute expressed sequence tags (ESTs) database (http://www.ncbi.nlm.nih.gov/nucest/?term=jute). Primers were designed according to these potential reference gene sequences using Primer 3 (http://bioinfo.ut.ee/primer3-0.4.0/primer3/) and based on the following criteria: GC content 45−80%, melting temperatures 58−62°C, primer lengths 18−24 bp, and amplicon lengths 100−250 bp (See Table 1 for detailed primer information).

**Table 1:** **Primer sequences and amplicon characteristics of the 11 candidate internal control genes**.

**Gene**	**Gene description**	***Arabidopsis* ortholog locus**	**Primer sequence F/R (5′–3′)**	**Product size(bp)**	**Efficiency (%)**	**R2**	**Mean Ct**	**SD**	**CV (%)**
18S *rRNA*	18S ribosomal RNA	AT3G41768	CTACGTCCCTGCCCTTTGTA	175	90.1	0.997	16.85	0.78	4.63
			GGTTCACCTACGGAAACCTTG						
*ACT*	Actin	AT3G12110	CATTACCATTGGGGCAGAAC	168	113.7	0.991	25.75	1.31	5.09
			GAGCCACCACTGAGGACAAT						
*ACT7*	Actin 7	AT5G09810	ACAATTGGAGCAGAGCGTTT	166	99.52	0.997	22.24	0.92	4.14
			TAGACCCACCGCTAAGCACT						
*DnaJ*	Chaperone	AT3G07590	TGTATGCACCGAGGAAAATG	154	104.1	0.999	22.22	0.75	3.38
	protein dnaJ		GTGGAAAAATCGTTGGCAAT						
*EF1*α	Elongation	AT1G07940	GAAGAAGGACCCATCTGGTG	130	104.2	0.990	18.28	0.94	5.14
	factor 1-alpha		TCCACAAAACCGCAATGTAA						
*RAN*	Ras-related small	AT5G59840	GCCATGCCGATAAGAACATT	167	99.52	0.996	26.37	1.29	4.89
	GTP-binding protein		GTGAAGGCAGTCTCCCACAT						
*TUB*α	Alpha-tubulin	AT4G14960	AATGCTTGCTGGGAGCTTTA	213	98.21	0.988	28.43	2.15	7.56
			GTGGAATAACTGGCGGTACG						
*TUB*β	Beta-tubulin	AT2G29550	CTGGTTCCTCTTCCTCACCA	201	108.9	0.999	22.40	1.07	4.78
			ACAAGATGTTCAGGCGTGTG						
*UBC*	Ubiquitin-conjugating	AT3G52560	CTGCCATCTCCTTTTTCAGC	150	108.6	0.992	21.20	1.13	5.33
	enzyme like protein		CGAGTGTCCGTTTTCATTCA						
*UBI*	Ubiquitin extension	AT2G47110	CCACTCTCCACCTTGTCCTC	158	108.9	0.989	21.44	0.62	2.89
	protein		CAGCCTCTGAACCTTTCCAG						
*UBQ*	Ubiquitin	AT5G20620	TCTTTGCAGGGAAGCAACTT	219	96.49	0.997	20.13	1.54	7.65
			CTGCATAGCAGCAAGCTCAC						
*WRKY15*	DNA-binding	AT2G23320	CTTGGACAGCGTTTTCTTCC	128	99.46	0.996	–	–	–
	Protein WRKY15		TGAATGGTTTTGGTGCAGAC						

*R^2^, regression coefficient; SD, standard deviation; CV, co-variance*.

To check the specificity of the amplicon, all primer pairs were initially tested via standard RT-PCR using the Premix Ex Taq (TaKaRa, Japan) and the amplification product of each gene was verified by electrophoresis on a 2% agarose gel. Real-time amplification reactions were performed with the Applied Biosystems 7500 Real-Time PCR System using SYBR® Premix Ex Taq™ (TaKaRa, Japan). Reactions were prepared in a 20 μl volume containing the following: 2 μl cDNA template, 0.4 μl of each amplification primer, 0.4 μl ROX Reference Dye II, 10 μl 2x SYBR Premix Ex Taq™, and 6.8 μl dH_2_O. Amplifications were performed with an initial 30 s step of 95°C followed by 40 denaturation cycles at 95°C for 5 s and primer annealing at 60°C for 34 s. The melting curve ranged from 60 to 95°C and temperature was increased in increments of 0.2°C every 10 s for all PCR products. ABI Prism Dissociation Curve Analysis Software was used to confirm the occurrence of specific amplification peaks. All qRT-PCR reactions were carried out in triplicate with template-free negative controls being performed in parallel.

### Statistical analyses of gene expression stability

To select a suitable reference gene, three publicly available software packages, geNorm (version 3.5), NormFinder and BestKeeper, were used to analyze the stability of each reference gene. All analyses using these packages occurred according to the manufacturers' instructions. For geNorm and NormFinder algorithms, the raw Ct values from the qRT-PCR were transformed into the relative expression levels using the following formula: E^−Δ*Ct*^, where ΔCt equaled each corresponding Ct value minus the minimum Ct value. Then, the relative expression values were imported into geNorm and NormFinder to analysis gene expression stability. For BestKeeper analysis, the Ct value was used as input data to calculate the coefficient of variation (CV) and the standard deviation (SD). The comprehensive rankings of the best reference genes were obtained by integrating the results of three algorithms. To validate the reliability of the selected reference genes, we analyzed the relative expression levels of the transcription factor *CcWRKY15* in all tested samples. Additionally, a standard curve was generated from a 10-fold dilution of cDNA in a qRT-PCR assay using Microsoft Excel 2003. The PCR efficiency (E) and the regression coefficient (*R*^2^) were calculated using the slope of the standard curve according to the equation E = [10^−(1∕*slope*)^ − 1] × 100%. All other multiple comparisons were performed using the statistical analysis software SPSS 22.0 (SPSS Inc., USA).

## Results

### qRT-PCR data of candidate reference genes

A total of 11 candidate reference genes, including 18S *rRNA, ACT, ACT7, DnaJ, EF1*α, *RAN, TUB*α, *TUB*β, *UBC, UBI*, and *UBQ*, were identified and assessed under abiotic stress (NaCl stress and drought stress), biotic stress (*Colletotrichum siamense*) and in different tissues in this study. For each gene, the specificity of the designed primers was verified using agarose gel electrophoresis and the subsequent presence of a single band with the expected size (Figure S1), and further confirmed by the presence of a single peak in the melting curve analysis, which was done prior to performing qRT-PCR (Figure S2). As described in Table 1, the amplicon size ranged from 130 to 219 bp. The PCR efficiency (E) was greater than 90% and varied from 90.1% (18S *rRNA*) to 113.7% (*ACT*), and the regression coefficient (*R*^2^) ranged from 0.988 (*TUB*α) to 0.999 (*DnaJ* and *TUB*β) (Table 1).

**Figure 1:**
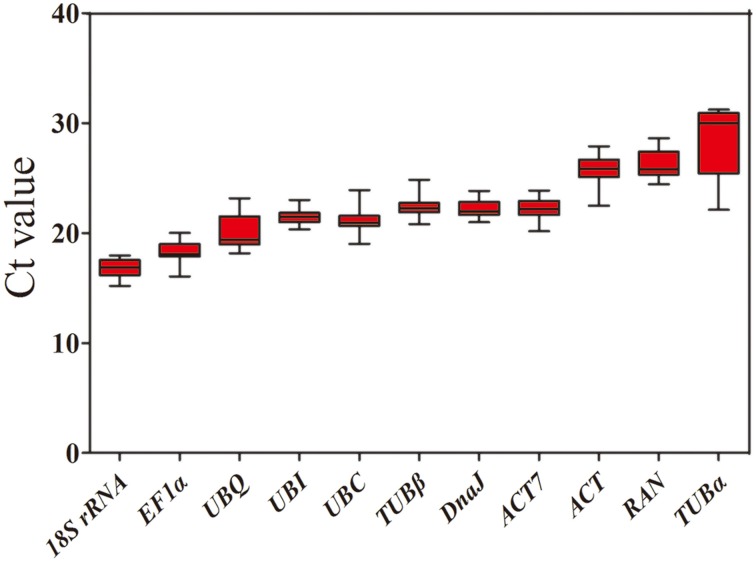
**Expression levels of 11 candidate reference genes across all experimental samples**. The box graph indicates the interquartile range, the median, and maximum/minimum values.

To evaluate stability of the reference genes across all experimental samples, the transcript abundances of the 11 candidate reference genes were detected by their mean Ct values. The Ct values of these candidates varied from 15.22 to 31.28, with the majority falling between 20.13 and 24.40 (Figure 1). Across all samples, 18S *rRNA* was the most abundantly expressed gene, with the lowest average Ct ± SD (16.85 ± 0.78), followed by *EF1*α (18.28 ± 0.94), *UBQ* (20.13 ± 1.54), *UBC* (21.20 ± 1.13), *UBI* (21.44 ± 0.62), *DnaJ* (22.22 ± 0.75), *ACT7* (22.24 ± 0.92), and *TUB*β (22.40 ± 1.07). *TUB*α was found to have the lowest level of expression of any of the genes tested, with a mean Ct ± SD of 28.43 ± 2.15, followed by *RAN* (26.37 ± 1.29) and *ACT* (25.75 ± 1.31) (Table 1). Small co-variance (CV) of the Ct value indicates that a given gene is more stably expressed. Among these 11 candidate reference genes, *UBQ* showed the greatest variation with CV value of 7.65%, whereas both *UBI* (2.89%) and *DnaJ* (3.38%) showed the least variation in their expression levels across all tested samples. The ranking of gene stability by CV was as follows: *UBI* > *DnaJ* > *ACT7* > 18S *rRNA* > *TUB*β > *RAN* > *ACT* > *UBC* > *TUB*α > *UBQ* (Table 1). Collectively, these results indicate that the transcript levels of the candidate reference genes varied across different experimental samples. Thus, it is essential to screen the most appropriate reference genes in jute in order to normalize gene expression analysis.

### Stability analysis of reference genes by genorm

**Figure 2:**
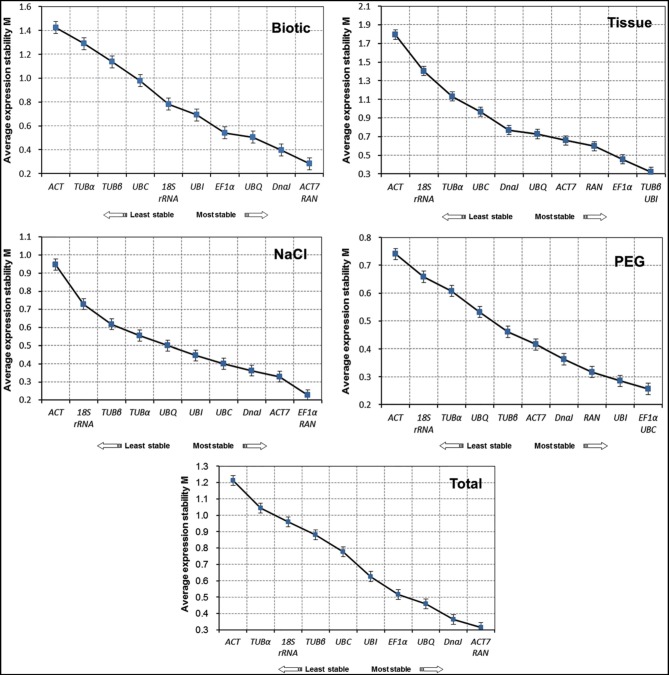
**Expression stability of 11 candidate genes in jute as calculated by geNorm**. Mean expression stability (M) was calculated following stepwise exclusion of the least stable gene in biotic stress samples, tissue samples, NaCl- and PEG-treated samples and all samples. The least stable genes are on the left and the most stable genes on the right.

A geNorm-based analysis was carried out to determine which candidate reference gene(s) would be optimal in each of the tested samples sets. As shown in Figure 2, genes were ranked according to their M values. Since a lower M value indicates increased stability, *RAN*, and *ACT7* were determined to be the most stable reference genes in total samples. In contrast, *TUB*α and *ACT* were the least stable reference genes. For each subset of the treatment, the top two reference genes for qRT-PCR normalization were *ACT7* and *RAN* in biotic stress subset, *TUB*β, and *UBI* in different tissues, *EF1*α and *RAN* for NaCl stress, and *EF1*α and *UBC* for drought stress. In general, the most stable genes across all experimental samples were *RAN* and *ACT7*, while *TUB*α, *ACT*, and 18S *rRNA* were the least stable (Figure 2).

**Figure 3:**
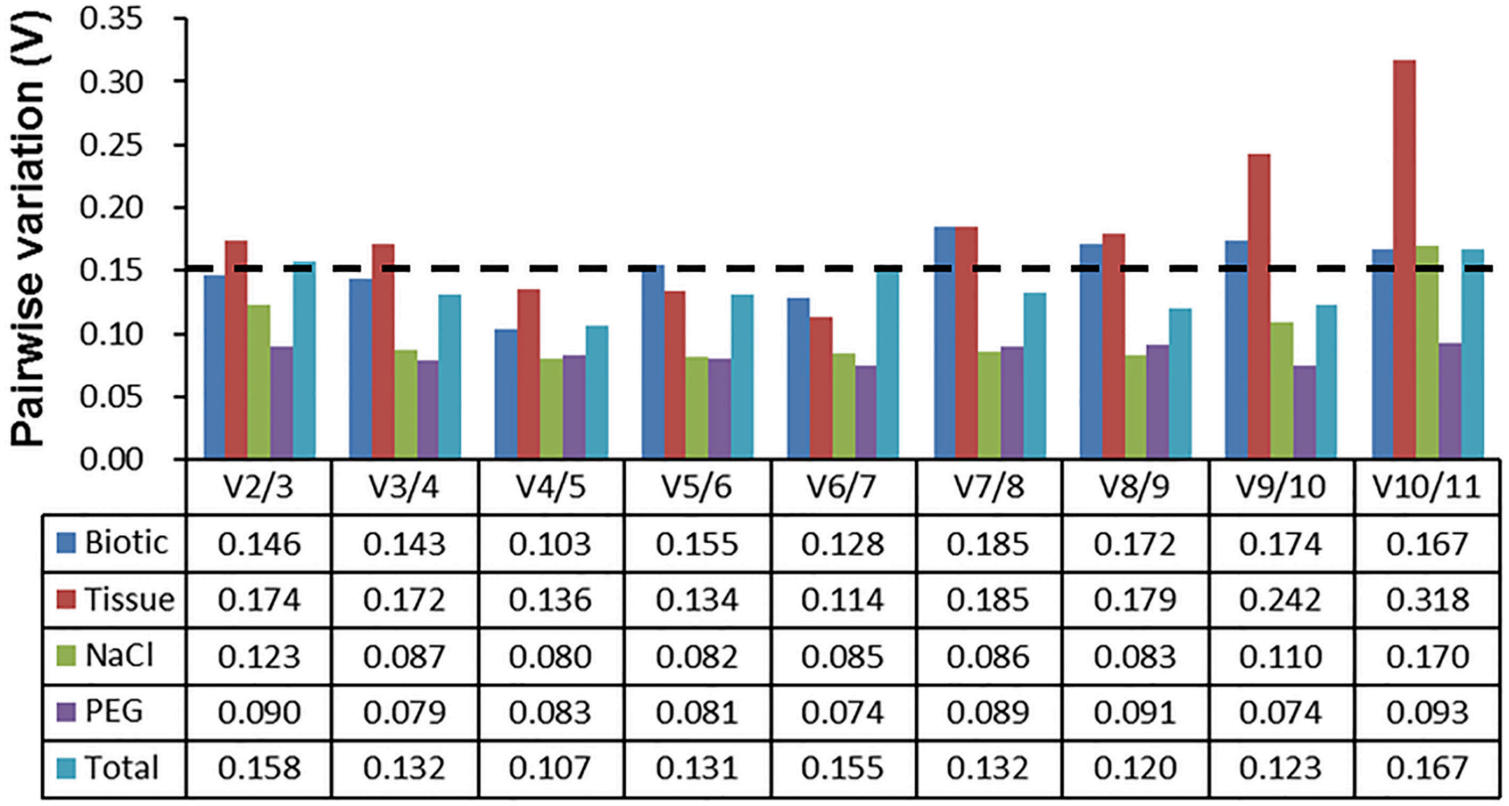
**Determination of the optimal number of reference genes for normalization by pairwise variation (V) using geNorm**. The pairwise variation (V_n_/V_n+1_) was calculated between normalization factors NF_n_ and NF_n+1_ by geNorm to determine the optimal number of reference genes for qRT-PCR data normalization.

The pairwise variation (V_n_) between normalization factors (NF_n_) calculated by the geNorm algorithm also determines the optimal number of reference genes for accurate normalization. A cut-off value of V_n∕n+1_ < 0.15 (Vandesompele et al., 2002) indicates that an additional reference gene makes no significant contribution to the normalization. As depicted in Figure 3, the V_2∕3_ values in biotic stress, NaCl stress, and PEG stress subsets were below 0.15, which indicated that two reference genes (*ACT7* and *RAN* for biotic subset; *EF1*α and *RAN* for NaCl subset; and *EF1*α and *UBC* for drought subset) were sufficient for accurate normalization. In the tissue subset, four reference genes (*TUB*β, *UBI, EF1*α, and *RAN*) were needed for accurate normalization, as the V_4∕5_ value was lower than 0.15. When the total samples were taken into account, the V3/4 value (0.132) was lower than the cutoff value of 0.15, which indicated that three genes (*ACT7, RAN*, and *DnaJ*) were suitable for all samples in this study (Figure 3).

### Stability analysis of reference genes by normfinder

The NormFinder approach was used to determine the stability of reference genes based on inter- and intra-group variance in expression. As shown in Table 2, similar results were generated by NormFinder, which predicted *ACT7* and *RAN* to be the two most stably expressed normalization factors in both biotic and total subsets. For the NaCl and PEG treated samples, *ACT7* and *RAN* performed as the best reference genes by the NormFinder analysis, while *ACT7* was ranked the second in NaCl subset, and *ACT7* and *RAN* the fifth and third in PEG subset by the geNorm analysis. For the tissue subset, the four most stable genes, *TUB*β, *EF1*α, *RAN*, and *UBI* determined by NormFinder were ranked the first, second, third and first by geNorm algorithm, respectively (Table 2; Figure 2).

**Table 2:** **Expression stability of candidate reference genes as calculated by Normfinder**.

**Rank**	**Biotic**		**Tissue**		**NaCl**		**PEG**		**Total**	
	**Gene**	**Stability**	**Gene**	**Stability**	**Gene**	**Stability**	**Gene**	**Stability**	**Gene**	**Stability**
1	*RAN*	0.142	*TUB*β	0.112	*ACT7*	0.040	*RAN*	0.053	*RAN*	0.116
2	*ACT7*	0.357	*EF1*α	0.126	*RAN*	0.167	*ACT7*	0.173	*ACT7*	0.262
3	*EF1*α	0.499	*RAN*	0.136	*UBQ*	0.190	*DnaJ*	0.206	*EF1*α	0.349
4	*DnaJ*	0.545	*UBI*	0.320	*EF1*α	0.236	*UBC*	0.262	*DnaJ*	0.410
5	*UBQ*	0.583	*ACT7*	0.383	*TUB*α	0.325	*EF1*α	0.264	*UBQ*	0.487
6	*UBI*	0.650	*DnaJ*	0.603	*UBC*	0.366	*UBI*	0.292	*UBI*	0.492
7	18S *rRNA*	0.734	*UBQ*	0.673	*DnaJ*	0.366	*TUB*β	0.376	*TUB*β	0.616
8	*TUB*β	0.869	*UBC*	0.846	*TUB*β	0.472	*UBQ*	0.404	*UBC*	0.660
9	*UBC*	0.882	*TUB*α	0.992	*UBI*	0.490	*TUB*α	0.543	*TUB*α	0.749
10	*TUB*α	1.079	18S *rRNA*	1.882	18S *rRNA*	0.744	18S *rRNA*	0.557	18S *rRNA*	0.830
11	*ACT*	1.257	*ACT*	2.409	*ACT*	1.288	*ACT*	0.702	*ACT*	1.262

### Stability analysis of reference genes by bestkeeper

The BestKeeper program was used to evaluate the stabilities of reference genes based on the coefficient of variance (CV) and the standard deviation (SD) of the average Ct values. The most stable genes were identified as those that exhibit the lowest CV and SD (CV ± SD), and genes with SD greater than 1 were considered unacceptable and should be excluded (Chang et al., 2012; Xiao et al., 2014). In the biotic stress subset, 18S *rRNA* (2.96 ± 0.51) and *UBI* (2.39 ± 0.52) had lowest CV ± SD values, and showed remarkably stable expression. In the NaCl- and PEG-treated subset, *TUB*α had the lowest CV ± SD values of 0.79 ± 0.24 and 0.96 ± 0.29, respectively, and showed the most stable expression. In the total samples subset, *UBI* (2.32 ± 0.50) and 18S *rRNA* (3.76 ± 0.63) were identified as the two best reference genes for normalization. These results are inconsistent with those acquired from the geNorm and NormFinder methods (Figure 2; Tables 2, 3). In the tissue samples subset, the most stable reference genes identified by BestKeeper were *UBI* (1.98 ± 0.42) and *TUB*β (2.03 ± 0.43). This result is consistent with the results obtained from geNorm and NormFinder analyses (Figure 2; Tables 2, 3).

**Table 3:** **Expression stability of candidate reference genes as calculated by BestKeeper**.

**Rank**	**Biotic**			**Tissue**			**NaCl**			**PEG**			**Total**		
	**Gene**	**SD**	**CV**	**Gene**	**SD**	**CV**	**Gene**	**SD**	**CV**	**Gene**	**SD**	**CV**	**Gene**	**SD**	**CV**
1	18S *rRNA*	0.51	2.96	*UBI*	0.42	1.98	*TUB*α	0.24	0.79	*TUB*α	0.29	0.96	*UBI*	0.50	2.32
2	*UBI*	0.52	2.39	*TUB*β	0.43	2.03	*UBC*	0.27	1.30	*DnaJ*	0.31	1.44	18S *rRNA*	0.63	3.76
3	*ACT7*	0.56	2.47	*DnaJ*	0.59	2.60	*UBI*	0.33	1.58	*UBC*	0.35	1.71	*DnaJ*	0.64	2.89
4	*DnaJ*	0.59	2.58	*RAN*	0.6	2.24	*RAN*	0.41	1.59	*UBI*	0.40	1.87	*ACT7*	0.72	3.22
5	*RAN*	0.61	2.18	*UBQ*	0.65	3.23	*UBQ*	0.43	2.25	18S *rRNA*	0.4	2.41	*EF1*α	0.72	3.92
6	*UBQ*	0.66	2.98	*UBC*	0.95	4.66	*EF1*α	0.44	2.41	*TUB*β	0.41	1.86	*TUB*β	0.78	3.48
7	*EF1*α	0.75	4.01	*TUB*α	0.97	4.11	*TUB*β	0.47	2.09	*UBQ*	0.42	2.17	*UBC*	0.81	3.81
8	*ACT*	0.86	3.25	*EF1*α	1.00	5.70	*DnaJ*	0.48	2.18	*ACT*	0.43	1.68	*ACT*	0.94	3.64
9	*UBC*	1.06	4.79	18S *rRNA*	1.03	6.28	*ACT7*	0.55	2.48	*RAN*	0.48	1.87	*RAN*	1.15	4.35
10	*TUB*α	1.13	4.32	*ACT7*	1.13	5.27	18S *rRNA*	0.59	3.43	*ACT7*	0.49	2.19	*UBQ*	1.32	6.54
11	*TUB*β	1.17	5.04	*ACT*	1.50	6.37	*ACT*	0.81	3.15	*EF1*α	0.56	3.09	*TUB*α	2.66	9.37

### Comprehensive stability analysis of reference genes

To acquire a consensus result of the best reference genes, three algorithms rankings of the stability were integrated, generating a comprehensive ranking according to the geometric mean of three rankings (Xiao et al., 2014). The comprehensive rankings were shown in Table 4: *RAN* and *ACT7* were ranked as the top two stable reference genes in the biotic stress subset, NaCl stress subset and total samples subset; *UBC* and *DnaJ* were the two most stable genes in the PEG stress subset; *TUB*β and *UBI* were the most stable genes, followed by *EF1*α and *RAN* in the tissue subset (Table 4). Taken the number of reference genes to use suggested by geNorm and the comprehensive rankings into consideration, the most stable and least stable combination of reference genes in each subset was shown in Table 5.

**Table 4:** **Expression stability ranking of the 11 candidate reference genes**.

**Method**	**1**	**2**	**3**	**4**	**5**	**6**	**7**	**8**	**9**	**10**	**11**
**(A) RANKING ORDER UNDER BIOTIC STRESS (BETTER-GOOD-AVERAGE)**											
NormFinder	*RAN*	*ACT7*	*EF1*α	*DnaJ*	*UBQ*	*UBI*	18S *rRNA*	*TUB*β	*UBC*	*TUB*α	*ACT*
geNorm	*ACT7*/*RAN*		*DnaJ*	*UBQ*	*EF1*α	*UBI*	18S *rRNA*	*UBC*	*TUB*β	*TUB*α	*ACT*
BestKeeper	18S *rRNA*	*UBI*	*ACT7*	*DnaJ*	*RAN*	*UBQ*	*EF1*α	*ACT*	*UBC*	*TUB*α	*TUB*β
Comprehensive ranking	*RAN*	*ACT7*	*DnaJ*	18S *rRNA*	*UBI*	*EF1*α	*UBQ*	*UBC*	*TUB*β	*ACT*	*TUB*α
**(B) RANKING ORDER UNDER DIFFERENT TISSUES (BETTER-GOOD-AVERAGE)**											
NormFinder	*TUB*β	*EF1*α	*RAN*	*UBI*	*ACT7*	*DnaJ*	*UBQ*	*UBC*	*TUB*α	18S *rRNA*	*ACT*
geNorm	*TUB*β/*UBI*		*EF1*α	*RAN*	*ACT7*	*UBQ*	*DnaJ*	*UBC*	*TUB*α	18S *rRNA*	*ACT*
BestKeeper	*UBI*	*TUB*β	*DnaJ*	*RAN*	*UBQ*	*UBC*	*TUB*α	*EF1*α	18S *rRNA*	*ACT7*	*ACT*
Comprehensive ranking	*TUB*β	*UBI*	*EF1*α	*RAN*	*DnaJ*	*UBQ*	*ACT7*	*UBC*	*TUB*α	18S *rRNA*	*ACT*
**(C) RANKING ORDER UNDER NACL STRESS (BETTER-GOOD-AVERAGE)**											
NormFinder	*ACT7*	*RAN*	*UBQ*	*EF1*α	*TUB*α	*UBC*	*DnaJ*	*TUB*β	*UBI*	18S *rRNA*	*ACT*
geNorm	*EF1*α/*RAN*		*ACT7*	*DnaJ*	*UBC*	*UBI*	*UBQ*	*TUB*α	*TUB*β	18S *rRNA*	*ACT*
BestKeeper	*TUB*α	*UBC*	*UBI*	*RAN*	*UBQ*	*EF1*α	*TUB*β	*DnaJ*	*ACT7*	18S *rRNA*	*ACT*
Comprehensive ranking	*RAN*	*ACT7*	*EF1*α	*TUB*α	*UBC*	*UBQ*	*UBI*	*DnaJ*	*TUB*β	18S *rRNA*	*ACT*
**(D) RANKING ORDER UNDER PEG STRESS (BETTER-GOOD-AVERAGE)**											
NormFinder	*RAN*	*ACT7*	*DnaJ*	*UBC*	*EF1*α	*UBI*	*TUB*β	*UBQ*	*TUB*α	18S *rRNA*	*ACT*
geNorm	*EF1*α/*UBC*		*UBI*	*RAN*	*DnaJ*	*ACT7*	*TUB*β	*UBQ*	*TUB*α	18S *rRNA*	*ACT*
BestKeeper	*TUB*α	*DnaJ*	*UBC*	*UBI*	18S *rRNA*	*TUB*β	*UBQ*	*ACT*	*RAN*	*ACT7*	*EF1*α
Comprehensive ranking	*UBC*	*DnaJ*	*RAN*	*UBI*	*EF1*α	*TUB*α	*ACT7*	*TUB*β	*UBQ*	18S *rRNA*	*ACT*
**(E) RANKING ORDER UNDER TOTAL SAMPLES (BETTER-GOOD-AVERAGE)**											
NormFinder	*RAN*	*ACT7*	*EF1*α	*DnaJ*	*UBQ*	*UBI*	*TUB*β	*UBC*	*TUB*α	18S *rRNA*	*ACT*
geNorm	*ACT7*/*RAN*		*DnaJ*	*UBQ*	*EF1*α	*UBI*	*UBC*	*TUB*β	18S *rRNA*	*TUB*α	*ACT*
BestKeeper	*UBI*	18S *rRNA*	*DnaJ*	*ACT7*	*EF1*α	*TUB*β	*UBC*	*ACT*	*RAN*	*UBQ*	*TUB*α
Comprehensive ranking	*ACT7*	*RAN*	*DnaJ*	*UBI*	*EF1*α	*UBQ*	18S *rRNA*	*TUB*β	*UBC*	*ACT*	*TUB*α

**Table 5:** **Best combination of reference genes based on the geNorm and comprehensive rankings in each subset**.

**Biotic**		**Tissue**		**NaCl**		**PEG**		**Total**	
**Most**	**Least**	**Most**	**Least**	**Most**	**Least**	**Most**	**Least**	**Most**	**Least**
*RAN*	*TUB*α	*TUB*β	*ACT*	*RAN*	*ACT*	*UBC*	*ACT*	*ACT7*	*TUB*α
*ACT7*		*UBI*		*ACT7*		*DnaJ*		*RAN*	
		*EF1*α						*DnaJ*	
		*RAN*							

### Reference genes validation

**Figure 4:**
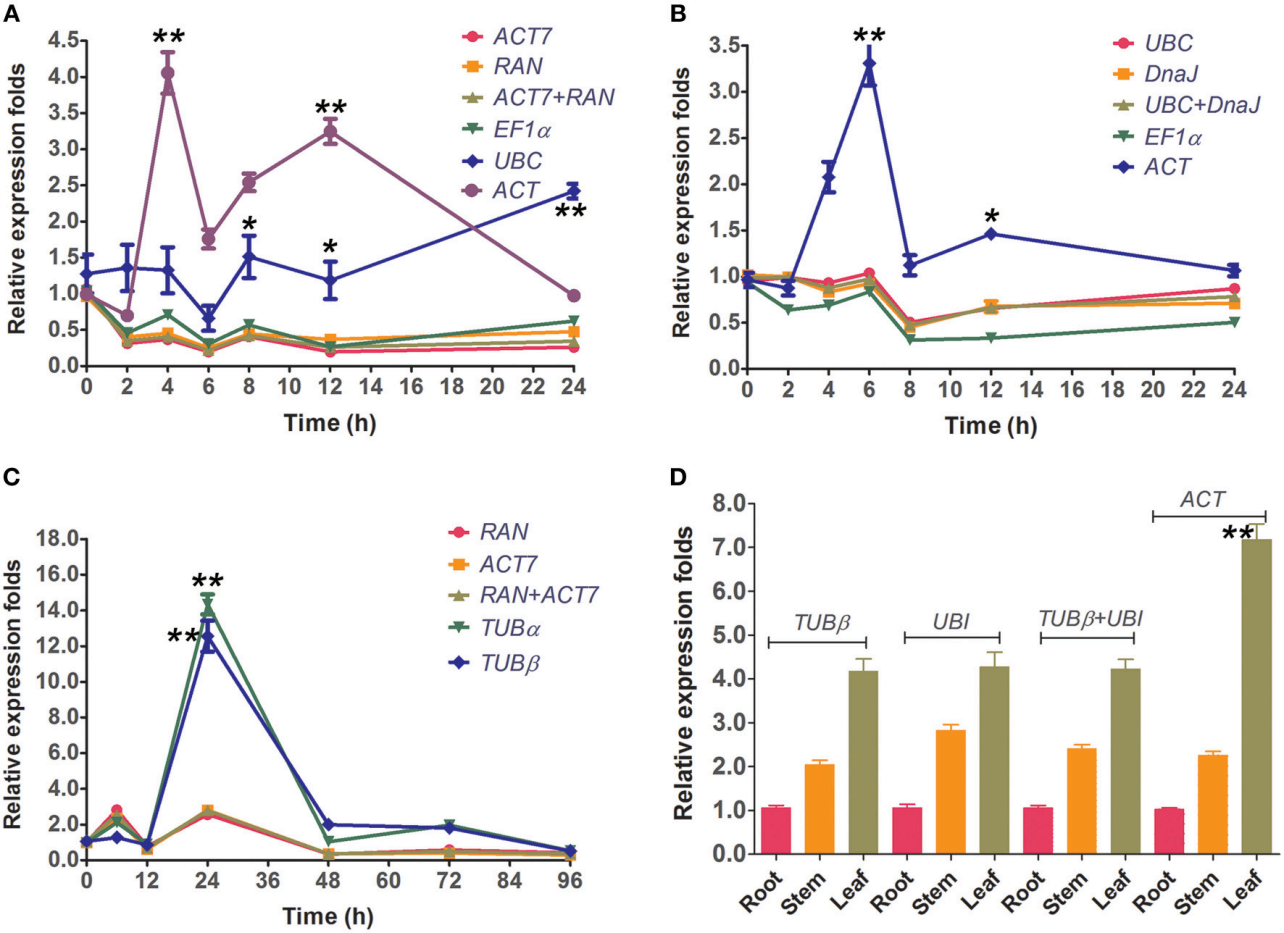
**Relative quantification of *CcWRKY15* expression using the validated reference gene(s)**. The results are represented as mean fold changes in relative expression when compared to the first sampling stage (0 h). cDNA samples were taken from the same subset used for gene expression stability analysis. **(A,B)** Leaves were collected from 3-week-old seedlings subjected to salt- and PEG-stress after 0, 2, 4, 6, 8, 12, and 24 h of treatment. **(C)** Leaves were collected from 3-week-old seedlings subjected to biotic stress (*C*. *siamense*) after 0, 6, 12, 24, 48, 72, and 96 h of treatment. **(D)** Different tissue types were collected from 3-week-old seedlings. ^*^ indicates statistically significant (*p* < 0.05); ^**^ indicates greatly statistically significant (*p* < 0.01).

To validate the selected reference genes, the relative expression levels of the target gene, *CcWRKY15* under different experimental conditions were evaluated using qRT-PCR. *CcWRKY15* is a homolog of *AtWRKY15*, which is known to be a central regulator in the response to oxidative stress and pathogenic infection (Vanderauwera et al., 2012). Thus, it would be expected to have similar expression patterns as *AtWRKY15* under abiotic and biotic stress conditions. However, a substantial divergence can occur in its relative transcript abundance when normalized to different kinds of reference genes. We therefore used the most stable reference genes found in each subset (*ACT7* and *RAN* for Biotic stress and NaCl stress; *UBC* and *DnaJ* for PEG stress) either singly or in combination, and the least stable reference gene (*ACT* or *TUB*α), to perform a qRT-PCR analysis. Results showed that in accordance with the behavior of *AtWRKY15* previously described in *A. thaliana*, the *CcWRKY15* functions as a negative regulator and was induced by salt-stress, oxidative-stress, and pathogenic infections (*C*. *siamense*) in jute (Figure 4). In addition, we also examined the reference gene (*EF1*α and *UBC* for abiotic stress; *TUB*β for fungal stress) selected by Ferdou et al. (2015) in NaCl stress, PEG stress, and fungal stress subsets. The results showed that *EF1*α could sever as a stable reference gene for normalization but *UBC* unstable under NaCl stress condition (Figure 4A). For the PEG stress subset, the relative expression folds of *CcWRKY15* normalized by *EF1*α were slightly decreased compared to the stable genes *UBC* and *DnaJ* (Figure 4B). For the biotic stress subset, reference gene *TUB*β was not as stable as that described by Ferdou et al. (2015), conversely, similar to the expression pattern of the worst gene *TUB*α (Figure 4C). Our tissue type analysis revealed that the transcript abundance of *CcWRKY15* was the highest in the leaf, followed by the stem and then the root (*p* < 0.01) (Figure 4D). Contrastingly, when the least stable reference gene was used as normalization factor, the expression level of *CcWRKY15* was significantly overestimated (*p* < 0.01). For example, the relative expression folds of *CcWRKY15* were approximately eight-fold (*p* < 0.01) higher than that of *ACT7, RAN*, or their combination at 4 h under NaCl stress, when *ACT* was used as normalization factor (Figure 4A).

## Discussion

There has been a surge in interest for environment-friendly materials and jute fiber—used as reinforcement component—has been widely used in the textile, papermaking, automotive, and aerospace industries. Given its popularity, the need for the application of new genomic tools has become increasingly important (Sydenstricker et al., 2003; Corrales et al., 2007). New technologies such as qRT-PCR now make it possible to understand the molecular mechanisms underpinning the commercially important traits of jute. qRT-PCR is an essential tool that can be used in studies of target gene expression patterns. Although past studies have used 18S *rRNA* as a reference gene for examining the functions of UDP-glucose pyrophosphorylase and caffeoyl-CoA 3-O-methyltransferase in jute (*C*. *capsularis*) (Zhang et al., 2013, 2014), and in the most recent study were 7 reference genes tested under abiotic and biotic stress in the other jute species *Corchorus olitorius* (Ferdou et al., 2015), little information is available on the systematic exploration and validation of a set of suitable reference genes in jute (*C*. *capsularis*). In addition, it has been reported that ribosomal RNA genes are not adequate reference genes due to their high transcription abundance. Ultimately, this could lead to experimental error when normalizing genes with weak expression (Jain et al., 2006; Niu et al., 2014). Comparing our results to that of Ferdou et al. (2015), the stability values of three out of seven reference genes (*EF1*α and *UBC* for abiotic stress; *TUB*β for biotic stress) were significantly lower in our experiment. For instance, when *UBC* was used as reference gene, *CcWRKY15* had a 5.0-fold higher expression value at 24 h under NaCl stress condition (Figure 4A); when *TUB*β as an internal reference, *CcWRKY15* had a 6.0 times higher at 24 h after inoculation (Figure 4C). Additionally, *TUB*β was found to be one of the least stable genes in our study. These expression stability differences might be a result of different species/cultivars analyzed in the compared experiment conditions. It is consistent with the previous studies that the stability of reference genes is not only cultivar/species specific but may also be tissue specific and influenced by the different experimental treatments (Nicot et al., 2005; Štajner et al., 2013; Zhuang et al., 2015). This is also the primary reason for validating the stability of reference genes for a specific genotype and/or experimental treatment.

In this study, we used three publicly available programs, geNorm (Vandesompele et al., 2002), NormFinder (Andersen et al., 2004), and BestKeeper (Pfaffl et al., 2004), to evaluate the expression stability of 11 candidate reference genes in a total of 25 jute samples taken from different tissues and experimental treatment groups. We found different rankings for the selected genes after comparison to the ranking of the candidates generated by the three algorithms (Figure 2; Tables 2, 3). This apparent divergence probably reflects the discrepancies in the three statistical algorithms to calculate stability. NormFinder takes the inter- and intra-group variations into account, and combines them into a stability value, and finally ranks the top genes with minimal inter- and intra-group variation. In contrast, geNorm identifies two reference genes with the highest degree of similarity in expression profile and the lowest intra-group variation (Andersen et al., 2004; Jian et al., 2008; Cruz et al., 2009; Liu et al., 2012). As for BestKeeper, this program determines the stability ranking of the reference genes based on the coefficient of variance (CV) and the standard deviation (SD) values. The most stable genes are identified as those that exhibit the lowest CV ± SD values (Chang et al., 2012; Xiao et al., 2014). Similar methods have been used in previous studies of different species, such as *Salicornia europaea* (Huang et al., 2014; Xiao et al., 2014), *Populus euphratica* (Wang et al., 2014), and *Cynodon dactylon* (Chen et al., 2014). Thus, referring to the previous studies (Štajner et al., 2013; Xiao et al., 2014), the integrated results were obtained from three programs, leading to a more comprehensive ranking and better accuracy for each gene (Table 4).

Of the top three reference genes defined by three algorithms in the total samples subset, *ACT7* and *RAN* were found to be the best candidates for the biotic stress and NaCl stress subsets. The strong performance of *ACT7* in jute (*C*. *capsularis*) was consistent with the results obtained in the developmental stage series of *G*. *max* (Jian et al., 2008), during grape berry development of *V*. *vinifera* (Reid et al., 2006), and across different tissues and cold-treated samples of *Platycladus orientalis* (Chang et al., 2012). However, this gene performed poorly in studies of *O*. *sativa* (Jain et al., 2006), *S*. *officinarum* (Ling et al., 2014), and *S*. *tuberosum* (Nicot et al., 2005), suggesting that the expression levels of reference genes are variable among different species. *RAN* was the other most stable reference gene found in the present study. It has also been shown to be the best performer across different tissue and hormone treatment samples of *M*. *acuminata* (Chen et al., 2011) as well as in the leaf or plant growth regulator treatment samples of *C*. *melo* (Kong et al., 2014). *DnaJ* showed relatively stable expression and ranked third across all samples (Figure 2; Tables 3, 4). This gene has also been recommended as the best reference gene in different tissues and NaCl-treated samples of *P*. *orientalis* (Chang et al., 2012).

However, the data presented show that the candidate reference genes *EF1*α, *UBC, UBI, UBQ*, and *TUB*β were moderately expressed and had variable rankings in this study. For example, *EF1*α was ranked first by geNorm analysis and was stably expressed in NaCl- and PEG-treated samples, but was shown to be the fourth in NaCl stress subset analyzed by NormFinder; the fifth in PEG stress subset by NormFinder and Comprehensive ranking. Previously, *EF1*α showed stable expression during abiotic and biotic stress in *O*. *sativa* (Jain et al., 2006), *L*. *chinensis* (Zhong et al., 2011), *S*. *tuberosum* (Nicot et al., 2005), and *C*. *sativus* (Wan et al., 2010), but was shown as an unsatisfactory reference gene in *T*. *aestivum* (Paolacci et al., 2009), *G*. *max* (Jian et al., 2008), *N*. *benthamiana* (Liu et al., 2012), and *P*. *orientalis* (Chang et al., 2012). *UBC*, an ubiquitin-conjugating enzyme gene, was ranked first in the PEG stress subset, which was also the optimal reference gene under NaCl- and PEG-treated conditions in *P. orientalis* (Chang et al., 2012) and *C. olitorius* (Ferdou et al., 2015); however, it was not the best choice for normalization in the different tissues of bamboo (Fan et al., 2013). *UBQ* were relatively weakly expressed across all the experimental samples according to the three programs and comprehensive analyses, but were the optimal reference genes for developmental stages and under NaCl- and PEG-treated conditions in *P*. *orientalis* (Chang et al., 2012). As previously noted, *TUB*β was the most stable reference gene across various developmental stages of *G*. *max* (Jian et al., 2008), leaf senescence system of *H*. *annuus* (Fernandez et al., 2011), and among different tissues and PEG-treated samples of *P*. *orientalis* (Chang et al., 2012). In this study, similarly, all of the algorithms ranked *TUB*β in the first position, indicating that *TUB*β was the optimal choice as an internal control gene for different tissues investigated in jute (*C*. *capsularis*). *UBI*, another stable reference gene, showed relatively small variation in tissues subset, which was also the most stable reference gene in different tissues of *C*. *sativus* (Wan et al., 2010). In different tissues subset, in addition to expression of *TUB*β and *UBI*, the expression of *EF1*α and *RAN* was also stable and therefore they were considered as the suitable reference genes (Figure 2; Table 4).

Interestingly, the commonly used reference genes *TUB*α, 18S *rRNA*, and *ACT* performed poorly and were not suitable for most of the experimental conditions. Several studies have shown similar results. For example, *TUB*α was found to be unstable as reference gene in the developmental stage in tomato (Expósito-Rodríguez et al., 2008) and in different flax tissue (Huis et al., 2010). 18S *rRNA* was considered as the least reliable reference gene in viral-infected *N. benthamiana* (Liu et al., 2012) and under different conditions in *C*. *sativus* (Wan et al., 2010). *ACT* was proved unsuitable for normalization in different flax tissues (Huis et al., 2010), during abiotic and biotic stress in *S*. *tuberosum* (Nicot et al., 2005), and in viral-infected *N. benthamiana* (Liu et al., 2012). Taken together, these findings indicate that large numbers of experimental data on gene expression should be acquired to investigate the transcript stability of commonly used reference genes under different experimental conditions.

To further validate the feasibility of the reference genes screened in this study, we analyzed the transcription profiles of the WRKY domain gene *CcWRKY15*, a homolog *AtWRKY15* of *A*. *thaliana*. *AtWRKY15* has been shown to be a key regulator of plant growth and salt/osmotic stress responses in *A*. *thaliana* (Vanderauwera et al., 2012). In this study, the expression of *CcWRKY15* was normalized using the most stable reference genes in each subset both singly and combined as well as a least stable gene as an internal control (Figure 4). Our results showed that expression of *CcWRKY15* was negatively induced by NaCl- and PEG-treated stress (Figures 4A,B) and was significantly increased after 24 h of inoculation treatment (Figure 4C) (*p* < 0.01). By comparing the expression pattern of *CcWRKY15* with that reported in *A*. *thaliana* (Vanderauwera et al., 2012), a supported result was found. Therefore, the results obtained from this study are credible. Moreover, we compared the results from the jute species *C*. *olitorius* selected by Ferdou et al. (2015) under the same conditions, the results showed great differences between them. These results underscore the fact that inappropriate utilization of reference genes without validation may generate bias in the analysis and lead to misinterpretation of qRT-PCR data.

## Conclusion

We present here a systematic attempt to validate a set of candidate reference genes for the normalization of gene expression using qRT-PCR in jute (*C*. *capsularis*) under abiotic (salt and drought) and biotic (*Colletotrichum siamense*) stress conditions as well as across different tissue types. The expression stability of the 11 candidates was analyzed by the three applications (geNorm, NormFinder, and BestKeeper), and their results were furtherly integrated into a comprehensive ranking based on the geometric mean. For gene expression study under biotic stress and NaCl stress, we recommend *ACT7* and *RAN* to normalize the qRT-PCR data. For gene expression study under PEG stress, *UBC*, and *DnaJ* are the two most suitable reference genes in *C*. *capsularis*. For the study of gene expression in the different tissues, *TUB*β, *UBI, EF1*α, and *RAN* are recommended as the best reference genes for normalization. In addition, the two least stable reference genes 18S *rRNA* and *ACT* should be carefully used for normalization. Furthermore, the feasibility of the reference genes screened was further confirmed by comparing the expression pattern between *CcWRKY15* and *AtWRKT15*, and the selected reference genes can perform significantly better in gene expression normalization. In particular, the reference genes selected in current study will facilitate the future work on gene expression studies in *C*. *capsularis*.

### Conflict of interest statement

The authors declare that the research was conducted in the absence of any commercial or financial relationships that could be construed as a potential conflict of interest.
