# Supplementary figures and images for: Selection of reliable reference genes for quantitative real-time PCR gene expression analysis in Jute (Corchorus capsularis) under stress treatments
Source: Front Plant Sci. 2015 Oct 14;6:848. doi: 10.3389/fpls.2015.00848 (PMC4604321; doi:10.3389/fpls.2015.00848)

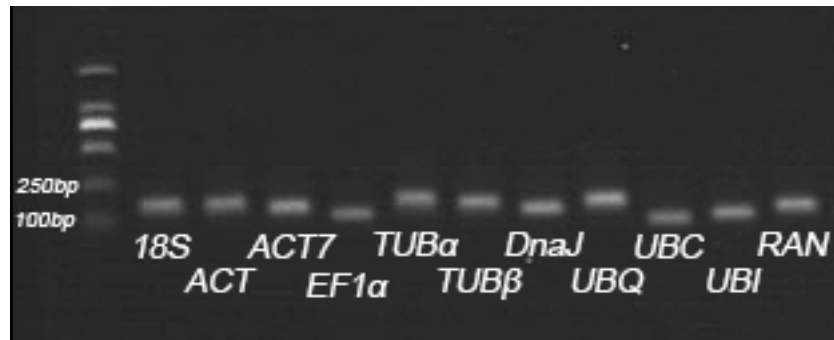

Fig. S1. PCR products of 11 candidate reference genes were checked on a 2.0% agarose gel.

Supplement: Supplementary file 1 [file Image1.PDF]

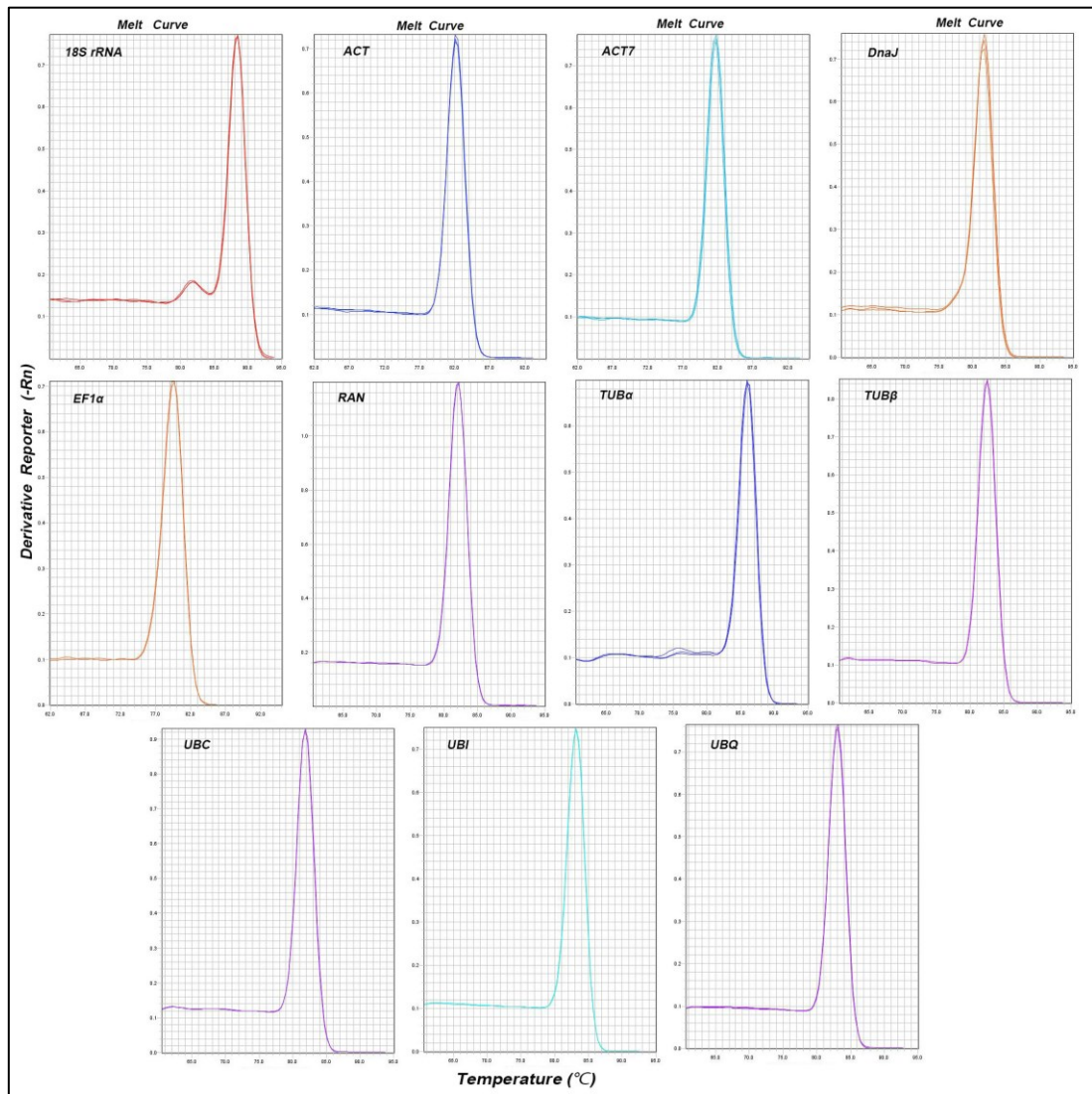

**Fig. S2. Melting curves of the 11 candidate reference genes tested in this study.**

Supplement: Supplementary file 2 [file Image2.PDF]
